# Highly stretchable large area woven, knitted and robust braided textile based interconnection for stretchable electronics

**DOI:** 10.1038/s41598-021-83480-x

**Published:** 2021-02-17

**Authors:** Min Ju Yun, Yeon Hyang Sim, Dong Yoon Lee, Seung I. Cha

**Affiliations:** 1 Energy Conversion Research Center, Electrical Materials Research Division, Korea Electrotechnology Research Institute, 12, Jeongiuil-gil, Seongsan-gu, Changwon, 51543 Korea; 2 Department of Electro-Functionality Materials Engineering, University of Science and Technology, 12, Jeongiuil-gil, Seongsan-gu, Changwon, 51543 Korea

**Keywords:** Electronic devices, Structural materials

## Abstract

With the rapid development of stretchable and wearable technologies, stretchable interconnection technology also demanded along it. Stretchable interconnections should have high stretchability and stable conductivity for use as an electrode. In addition, to develop to commercialization scale from research scale, a simple fabrication process that can be scaled up, and the stretchable interconnection should be able to be electrically connected to devices or modules directly. To date, printable conductor inks, liquid metals and stretchable structured interconnections have been reported for stretchable interconnections. These approaches have demonstrated high stretchability and conductivity, but in aspect of scale, it is appropriate to apply in micro-scale devices. For requirements of stretchability, conductivity and direct integration into meso- or centimeter-scale electronic devices or modules, here we introduce stretchable interconnections with a textile structure composed of metal fibers. The stretchable woven and knitted textiles show 67% strain and stable conductivity, and the cylindrical textile shows more than 700% strain with high strength. The stretchable textiles were fabricated using a weaving, knitting and braiding machine that can be used to produce textiles without any limit to length or area. These textiles exhibit high and stable conductivity even under deformation, and can be directly integrated into devices or modules by soldering. These high-performance stretchable textiles have great potential for commercial applications.

## Introduction

Further development of stretchable and wearable devices, or rigid devices and modules on a stretchable substrate, requires development of stretchable interconnection technology that can endure deformation from an external environment^1–6^.

Research on stretchable interconnections between micro-scale devices to date has used printable conductive inks^7–14^, liquid metals^15,16^ and stretchable structured interconnections^17–28^. Printable conductive inks are viscous inks that have been formulated by combining conductive materials and stretchable elastomers. Conductive fillers or particles such as metal nanoparticles (NPs), silver (Ag) nanowires (NWs), copper (Cu) NWs or carbon nanotubes (CNTs) are mixed with a stretchable polymer such as thermoplastic polyurethane resin (TPU), polydimethylsiloxane (PDMS) or silicone rubber^8,10,11^. The ink can then be directly patterned on a stretchable substrate and can be scaled up using other printing or ink-jet methods. It is necessary to optimize the concentrations of the conducting material and the stretchable polymer to strike a balance between the conductivity and the stretchability of the interconnection. In this method, conducting connection path should be considered during stretching because the polymer in the mixture is an insulator. By contrast, methods that utilize liquid metal show high conductivity even under deformation, and high stretchability. In this technique, liquid metal is filled into a stretchable elastomer though a microfluidic approach, and patterning is also possible. In micro-scale structured stretchable interconnections include spring-like^17^, mesh^18,19^, kirigami or origami ^20–23^ and wavy designs^24,25^, and exhibit high stretchability and conductivity with structure’s nature. However, these designs utilize a large surface area for one electrode. There are also limitations in scaling up to long lengths and large areas because these structures are fabricated using micro processing methods such as sputtering, lithography and etching, which work at sizes of under 8 inches.

For stretchable interconnection in meso- or centimeter-scale devices or modules, fiber or textile structure with stretchable encapsulation materials ^28–30^ and metal materials like foil or mesh with stretchable structure were used ^31,32^. These kinds of interconnection also limited in long length, continuous fabrication process and using actual production machine.

Hence, high-performance continuous process is required that enables scale-up of highly stretchable interconnections for meso- or centimeter-scale devices or modules with stable conductivity. Here, we introduce a stretchable interconnection using a textile structure composed of metal fibers. Using the metal fibers, three types of stretchable textile, including a rotated woven textile, a knitted textile and a cylindrical textile were fabricated using a weaving, knitting and braiding machine. High stretchability can be achieved by creating an open loop or a rhombus shape in the space between the intersections of fibers in the textile, and the textiles exhibit high conductivity and zero resistance loss when used as an interconnection. The stretchable woven and knitted textile interconnections showed 67% strain and no loss of resistance when integrated with devices on a substrate. We also fabricated a stretchable cylindrical textile that exhibited a strain of greater than 700% and highly stable conductivity after 500 cycles of repetitive stretching at a strain of 500%. The machine fabrication technique used for these textiles can be scaled up in both length and area, and the textiles can be simply and directly integrated with devices or modules by soldering. Hence, these textiles have great potential for applications in commercial electronics.

## Results and discussion

**Figure 1:**
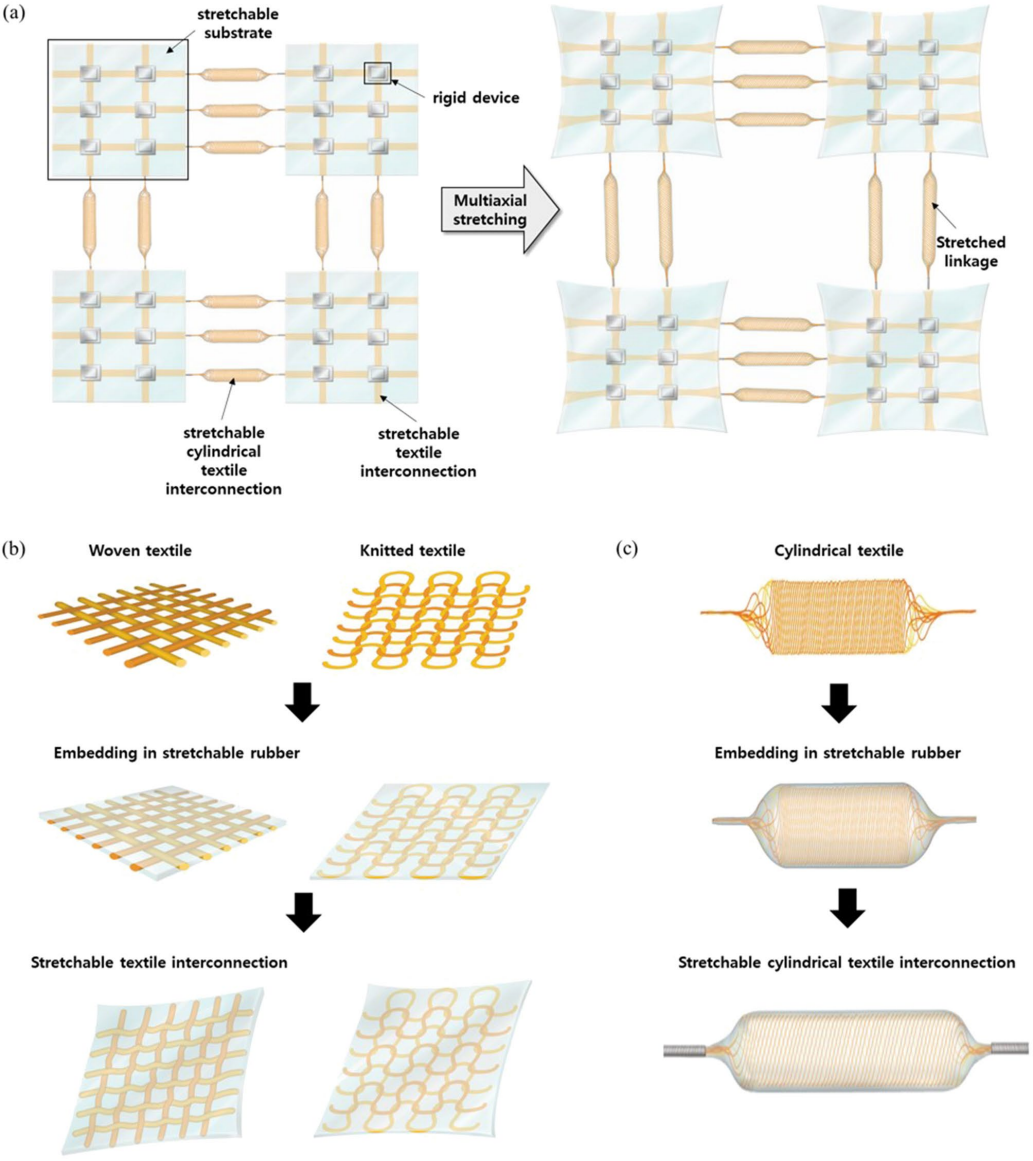
(**a**) Schematic image of rigid islands interconnected with stretchable textile interconnections and cylindrical interconnections, and stretched multiaxially across the entire module. Schematic image of stretchable (**b**) woven and knitted textiles (**c**) cylindrical textile encapsulated in silicone rubber and stretched multiaxially.

Generally, rigid devices have better performance than flexible devices. It is possible to add other parts such as stretchable interconnections and substrates to rigid devices so that a module can absorb external forces and be transformable and recoverable along any deformation axis. Addition of stretchable conductors as interconnects between rigid devices on a stretchable substrate is a key approach to creating stretchable electronics. Significant research has been conducted on embedding or mixing conductor materials with stretchable polymers, and on fabrication of stretchable structured conductors with various designs. These exhibit high stretchability and conductivity, however the fabrication process for the stretchable interconnection, and integration with the device are generally separate steps in research lab level. Here, we introduce stretchable textile interconnections that exhibit better stretchability and conductivity and can be integrated with a device directly by soldering. As shown in Fig. 1a, we fabricated and applied three types of stretchable textile interconnection that can provide electrical connections between electronic devices or modules. For interconnection between devices within a single substrate, the stretchable woven textile and the knitted textile shown in Fig. 1b were used. For interconnection between modules over a greater distance, the stretchable cylindrical textile shown in Fig. 1c was applied. All three types of stretchable textile were fabricated using a weaving, knitting and braiding machine and encapsulated in silicone rubber.

**Figure 2:**
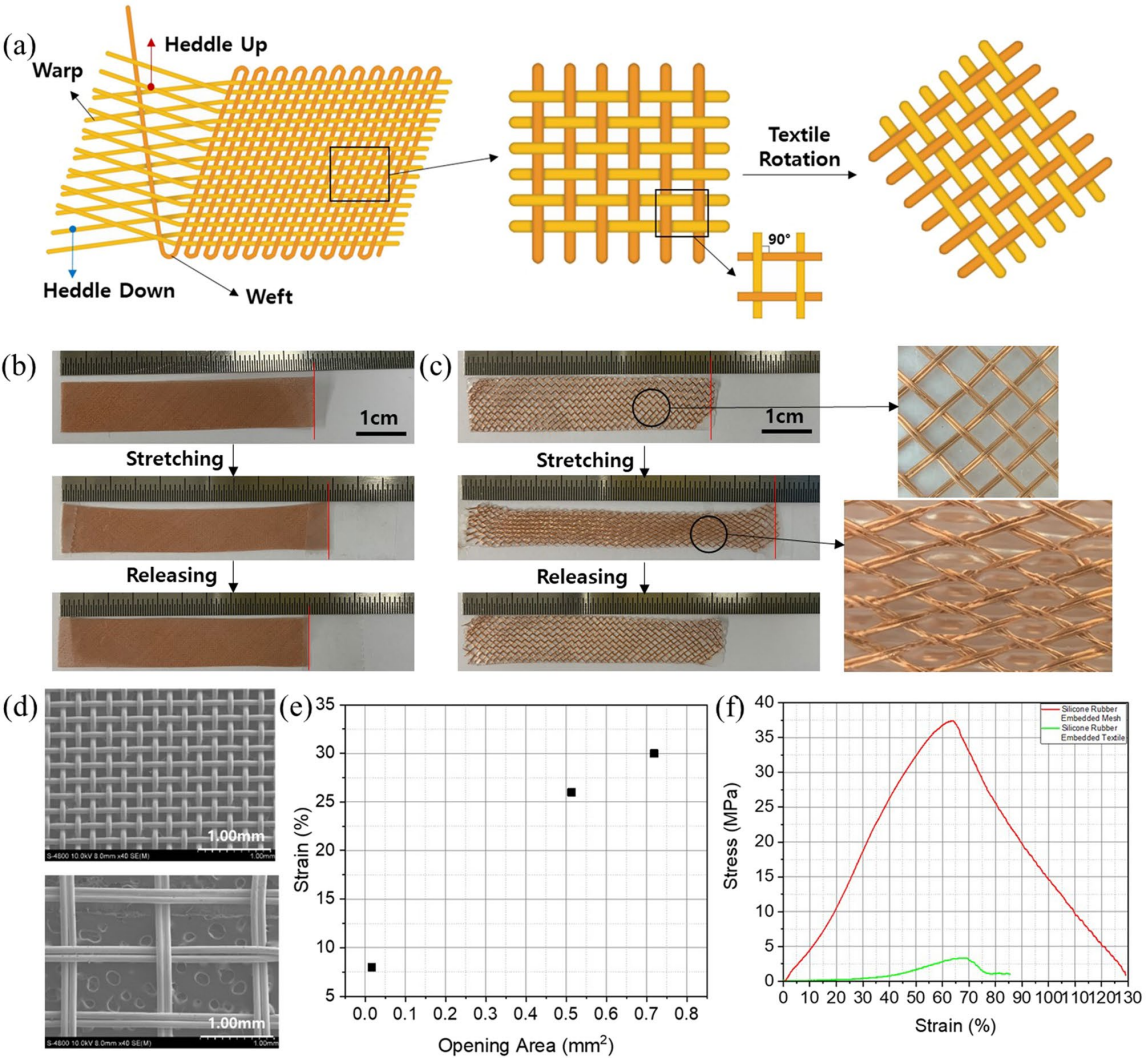
(**a**) Schematic images of the weaving process and textile rotation for the stretchable textile interconnection. Stretching and recovery photograph for the (**b**) commercial Cu mesh and (**c**) woven textile fabricated using the Jacquard weaving machine. The images on the right are enlarged images of the textile structure before and during stretching. (**d**) Scanning electron microscope images of commercial Cu mesh and wide woven textile. Relationship between (**e**) the area of the space between intersections and the strain and (**f**) the strain and stress of the Cu mesh and the woven textile.

The woven textile was fabricated using a Jacquard weaving machine with a large number of pistons for moving the warps. The warps connected to the pistons were raised by the textile patterns, and the warp yarns were intersected by the weft to create a woven textile as shown in Fig. 2a. In this textile, the intersecting warp and weft were initially at 90° to each other, and the space between these intersections had a square or rectangular shape. Stretchability in the textile was formed by rotating the entire textile by 45° to create a rhombic shape in the space between the intersections. When the space between the fiber intersections has a square or rectangular shape, the gripping force at the intersection points prevents the textile from stretching. However, if the space has a rhombic shape, there is limited gripping force at the intersection point when the textile is stretched along the x- or y-axis. And exact rotation 45° can give highest stretchability by forming longest cross-line length than other degrees as shown in Figure S1.

An important factor for controlling the stretchability is the shape of the space between fiber intersections during stretching deformation. We first measured a commercial copper mesh with a very small space between intersections, and confirmed its stretchability by pulling the mesh by hand; a very small strain of 8% was observed (Fig. 2b). We then fabricated a woven textile with a normal and large space area between fiber intersections, by controlling the space between warps. For the normal woven stretchable textile exhibited 26% strain, and for the wide one, it 30% strain when stretched by hand as shown in Fig. 2c and S2. The large rhombic area between intersections led to better strain as this textile was able to stretch sideways by changing rhombic space shape into a long rhombic shape by longer side and narrower top and bottom side under a small stress force, as shown as schematic illustration (Fig. S3). We analyzed the relationship between the space area between intersections and the strain under stretching by hand. The exact area of the space between the intersections was measured using scanning electron microscopy (SEM) (Fig. 2d), and it was confirmed that the strain increased almost linearly with an increase in the area of the space between the intersections (Fig. 2e). To confirm the tensile strength properties, we evaluated the relationship between the stress and the strain as shown in Fig. 2f. The stretchable woven textile with the largest area between intersections exhibited 67% strain, however, this textile had very poor strength as it had only a small number of intersections between warps and wefts. After peak stress, edge part of woven interconnection start releasing from woven structure, which results in dropping of stress. The second type of stretchable textile interconnection was a knitted textile, fabricated by forming loops in the course and wale directions, as shown in Figure S4. The knitted textile had stretchability through movement of the intersection points between loops within the height of the loop. The limit of the strain is determined by the loop height, which is determined by the movement distance of the fabrication machine, because the metal fibers themselves are not elastic over long strain range.

**Figure 3:**
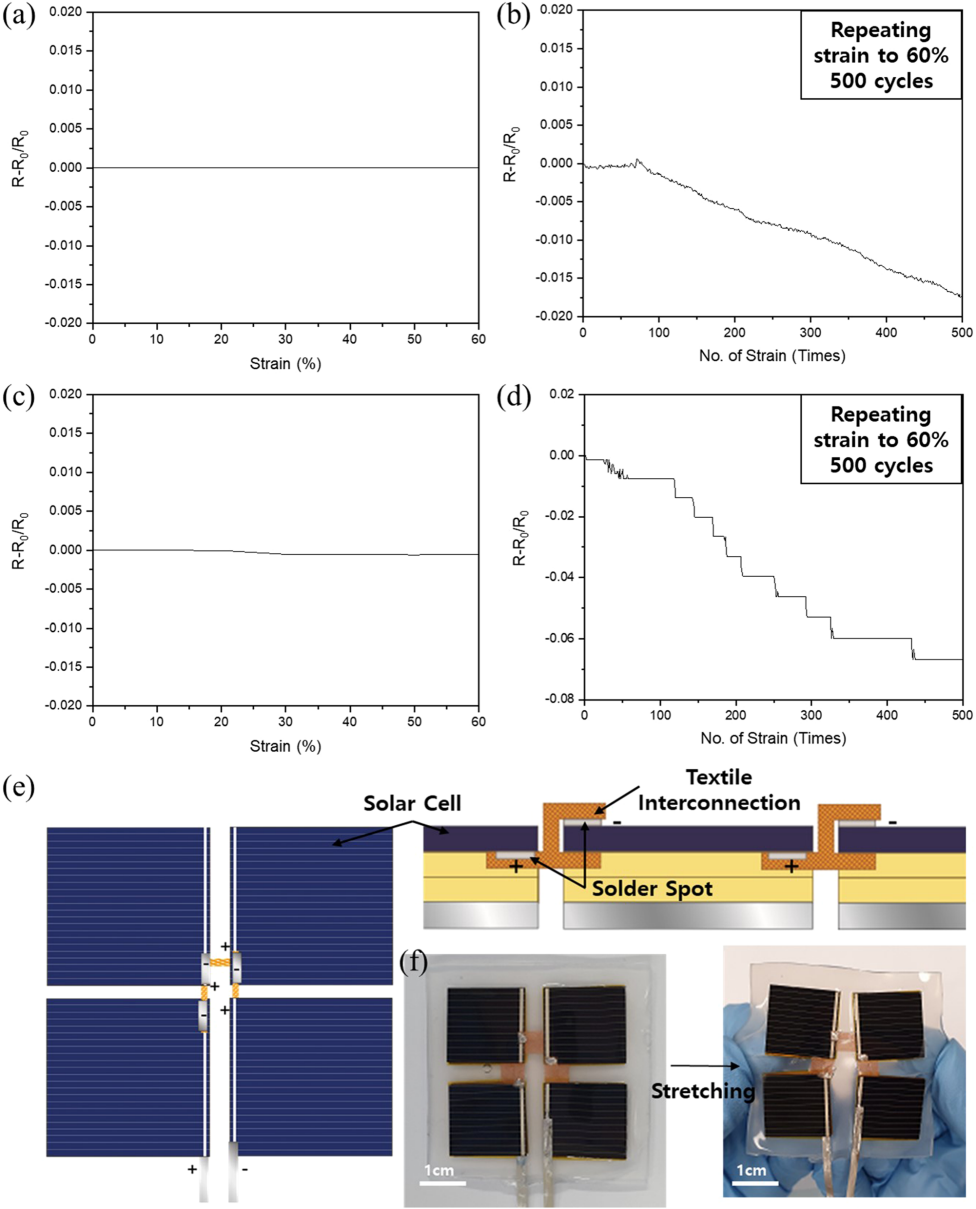
Electrical resistance of (**a**), (**b**) commercial Cu mesh and (**c**), (**d**) wide woven textile. Resistance was measured during strain to 60% (**a**), (**c**) and during 500 cycles of repetitive stretching to 60% strain (**b**), (**d**). (**e**) Four solar cells connected in series using the stretchable textile interconnection and (**f**) images before and during stretching.

To evaluate the performance of the textiles during electrical connection between devices, we measured the resistance change during deformation. The resistance was measured after stretching to 60%, and then after repeated stretching to 60% for 500 cycles to confirm the stability of the conductivity (Fig. 3a–d). For the woven textile both commercial mesh textile and wide-woven textile, show very stable electrical performance during stretching with not changing in resistance as shown in Fig. 3a,c. And for the repetitive deformation, both textile show decreasing resistance. Woven textile is created through intersection warp and weft yarns so conductivity is also transmitted in both directions. For the wide-woven textile, it shows larger resistance change than commercial woven mesh as shown in Fig. 3b,d. Wide-woven textile is created with large space area and smallest intersection points between warps and wefts so it is liable to change the form with less number of yarns. With some form of change under large mechanical deformation, surface temperature shows slightly unstable as well (Fig. S5). However, considering the order of resistance change, it shows stable electrical connection performance. We applied the stretchable textile as an electrical connection between silicon (Si) solar cells. Four Si solar cells were connected in series then encapsulated in silicone rubber, as shown in Fig. 3e. Here the stretchable textile acted not only as an interconnection but also as a bridge between cells. A stretchable module was fabricated and it was demonstrated that the performance was maintained under stretching deformation (Fig. 3f and Fig. S6). The interconnection performance was confirmed by using the textile as an interconnection for solar cell devices in an actual substrate, demonstrating its feasibility in a real-life application.

**Figure 4:**
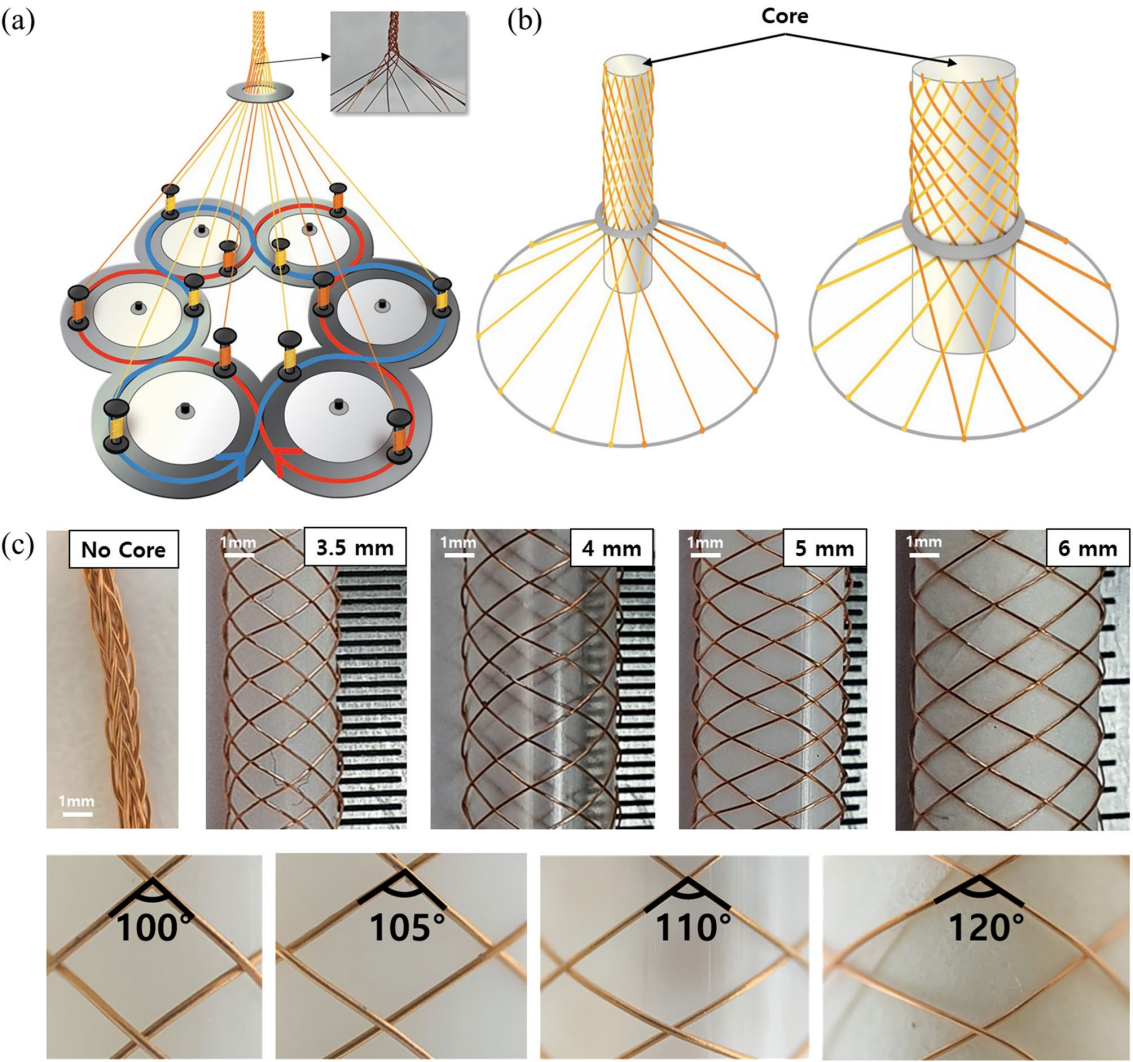
(**a**) Braiding technique using 12 Cu wires with rotation in two directions (clockwise, counterclockwise). (**b**) The rotated fibers show different intersection angles between the fibers depending on the core diameter. (**c**) Photographs of the cylindrical textile and the intersection angle with various core diameters (0 to 6 mm).

For interconnection between modules, we fabricated a stretchable cylindrical textile with better stretchability and strength than the woven textile. The cylindrical textile was fabricated using a braiding technique, by rotating one group of fibers clockwise (Fig. 4a, blue line) and the other group of fibers counterclockwise (Fig. 4a, red line), crossing each other along a central axis. By rotation, each yarn crosses over on below one other yarn, diamond braid structure was utilized in this textile. Through repeating this unit, cylindrical textile fabricated around central core^33,34^. The fiber rotation mechanism used to fabricate the textile produced a rhombic space where the fibers intersected, creating a perfect structure for a highly stretchable textile interconnection. In a stretchable cylindrical textile, the stretchability is determined by the size of the area between intersections, which is controlled by the angle between intersecting fibers. It can be stretched until the angle is close to 0° (Fig. S7). The fiber intersection angle can be controlled through the fiber rotation speed, the number of fibers and the core diameter around which the fibers are wound. Among these factors, the core diameter is the critical factor affecting the fiber intersection angle. Under identical conditions, a small core diameter will create a large slope of the rotating fibers and a small intersection angle, meaning that the cylindrical textile becomes more compact (Fig. 4b). As shown in Fig. 4c, cylindrical textiles were fabricated with no core, and with core diameters of 3.5, 4, 5 and 6 mm. For the fiber with no core, the intersection angle between fibers was very small and a very dense cylindrical textile was formed with almost no space between intersections. With increasing core diameter, the intersection angle between fibers increased (3.5 mm = 100°; 4 mm = 105°; 5 mm = 110°; and 6 mm = 120°), and the area between intersections also increased.

**Figure 5:**
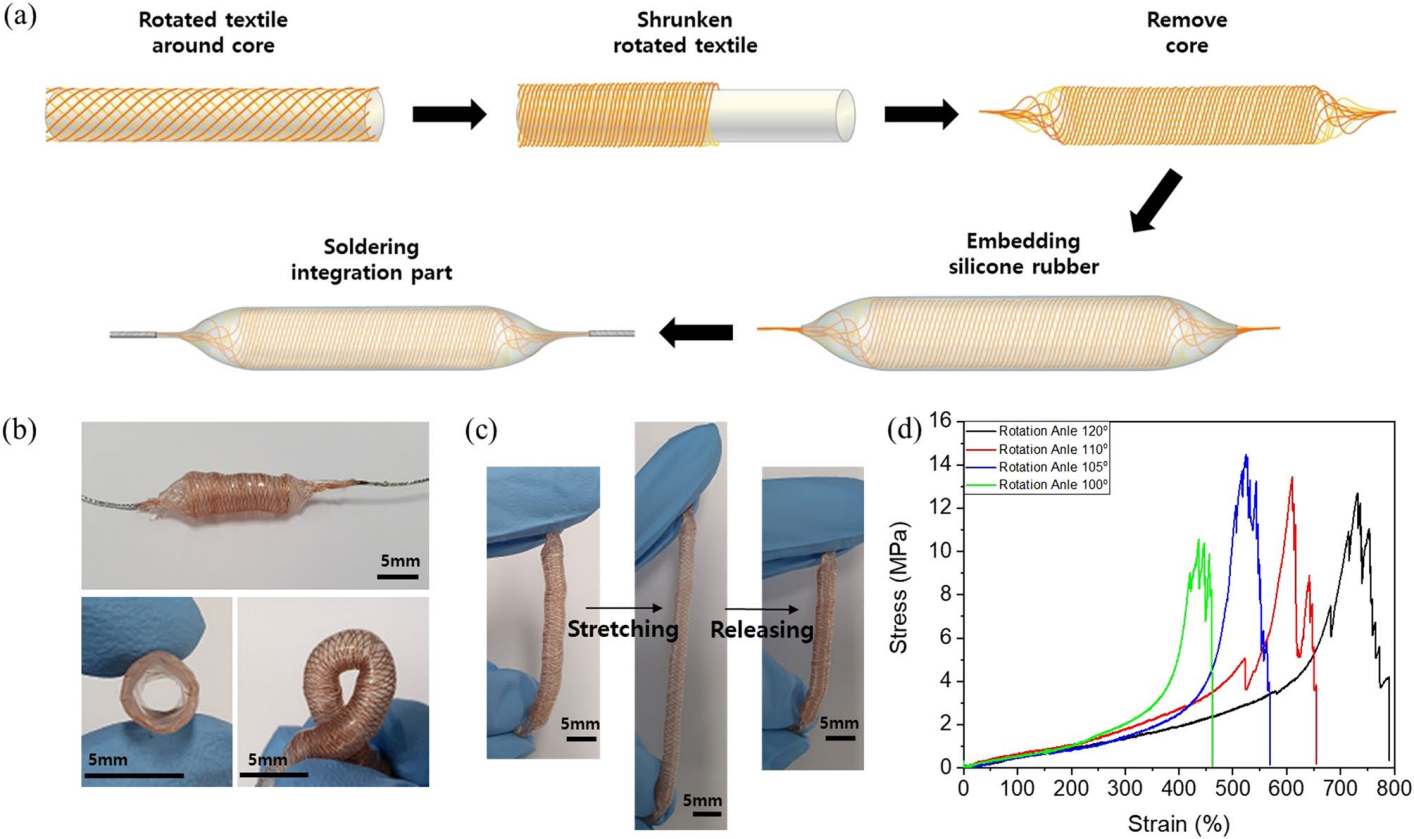
(**a**) Schematic of the fabrication process for the stretchable cylindrical textile interconnections from rotation of fibers around the core to embedding in silicone rubber and soldering edge parts for integration. (**b**) Photographs of stretchable cylindrical textile interconnection plane, cross section and twisted state and (**c**) stretching and recovery. (**d**) Relationship between strain and stress for the stretchable cylindrical textile interconnection depending on the intersection angle between fibers.

The fabrication process for the stretchable cylindrical textiles from braiding the fibers to encapsulation is shown schematically in Fig. 5a. First, a cylindrical textile was braided by fiber rotation around the core; then the textile was compressed to reduce the space between intersections as much as possible after the core was removed (Fig. S8). Then, the compressed cylindrical textile was encapsulated by dipping and rolling in mixed silicone rubber and encapsulation layer was formed surrounded cylindrical with uniform thickness (Fig. S9). This textile can be simply integrated into devices and modules by soldering at each end. We confirmed that the stretchable textiles were fabricated as a cylindrical shape and soldered perfectly, as shown in Fig. 5b. This cylindrical textile has excellent performance in stretching and recovering and also in twisting, and it is possible to manufacture it using a continuous process, so there is no limitation on the length or diameter as shown in Fig. 5c and Figure S10.

The tensile strength of the cylindrical textiles was measured to confirm the stretchability performance (Fig. 5d). The textiles exhibited excellent stretchability due to the rhombus-shaped space between intersecting fibers. The textile with the largest intersection angle of 120° exhibited the highest strain of more than 700%, and the textile with the 100-degree intersection angle had the lowest strain of 460%, and the mechanical parameters of tensile strength performance are shown in Table 1. All of the stretchable cylindrical textiles exhibited strain values greater than 500%, and hence can be considered to have excellent performance as stretchable textile interconnections.

**Table 1:** Mechanical performance of the stretchable cylindrical textile interconnection for each intersection angle between fibers. The values in the table are average ones measured from 6 samples with same process. The number in parenthesis is standard deviation value.

Intersection angle	Elastic constant (MPa) (SD)	Max strain (%) (SD)	Max stress (MPa) (SD)
100°	0.44 (0.03)	464.67 (11.59)	7.44 (0.39)
105°	0.41 (0.01)	555.33 (12.68)	9.06 (0.07)
110°	0.507 (0.02)	638.28 (13.95)	11.50 (0.48)
120°	0.518 (0.07)	762.33 (19.60)	11.21 (1.04)

SD, Standard Deviation.

**Figure 6:**
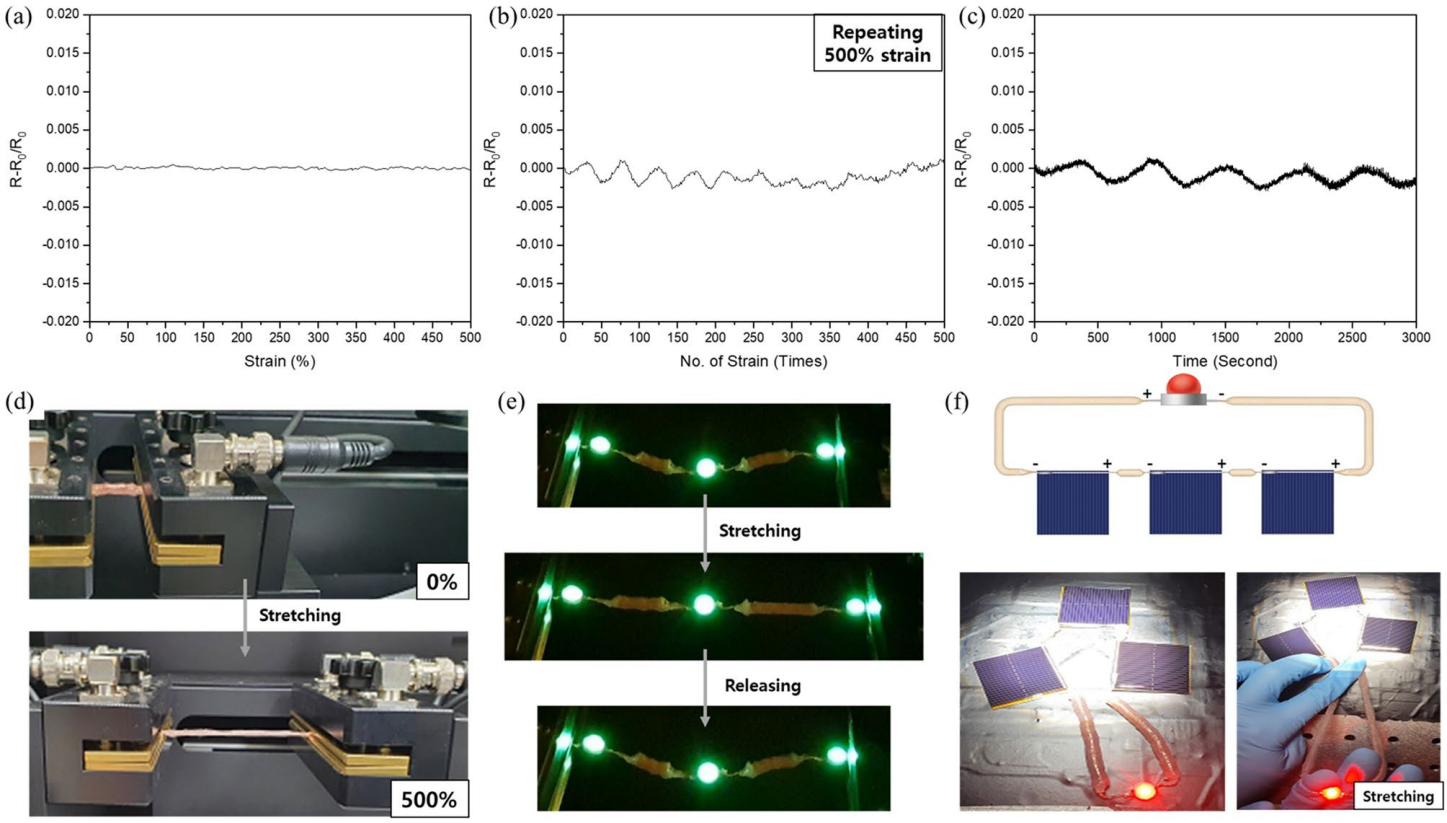
Measurement of the electrical resistance of the stretchable cylindrical textile interconnection during (**a**) strain to 500%, (**b**) 500 cycles of repetitive stretching to a strain of 500% and (**c**) repetitive stretching and recovery for 50 min. (**d**) Photographs of stretchable cylindrical textile interconnection before stretching and at 500% stretched state. LED lamps (**e**) integrated using the stretchable cylindrical textile interconnection during stretching deformation and (**f**) connected to a solar module.

The change in resistance was measured when the cylindrical textiles were stretched to 500%, and it was confirmed that there was almost no change in resistance. This confirmed stability indicates that the cylindrical textiles can be used as an interconnection between electronic devices or modules (Fig. 6a). In Fig. 6b, It shows resistance change during 500 cycles repeated stretching strain to 500%. Cylindrical textile When it recovered to 0% after strain to 500%, the resistance value was measured as one cycle. During stretching deformation 500 times, it shows stable electrical performance without change in resistance. And there's periodic changes every 50 cycles until repeating 250 times. To analysis periodic change, we should confirm the continuous resistance change data according to time which means whole cycle from stretching to recovery as shown in Fig. 6c and there's periodic change every 560 s. During 560 s, 35 times stretching to recovery repetitive deformation were conducted. After 35 repetitions, the stretchable cylindrical textile did not recover completely to original shape after stretching, and there's some shape deformation. So with that shape deformation resistance was little bit increased with deformation and then decreased with stable deformed shape. With stable changed shape, resistance value retuned to initial value, and then shape deformation and stabilization are repeated so showing a relationship with certain period. Comparing electricity performance to rotated woven textile interconnection, cylindrical textile interconnection shows much more stable characteristic.

A photograph of the stretchable cylindrical textile is shown in Fig. 6d, which indicates that the textile can be stretched without any damage even under stretching to 500%. The stretchable cylindrical textile was connected to three light emitting diodes (LEDs), and it was observed that even during recovery after stretching, the electrical contact was maintained and the LEDs remained on, even under repeated deformation (Fig. 6e). Three solar cells and an LED were integrated and electrically connected using the stretchable cylindrical textile, and the LED was turned on using the solar cells as the energy source, as shown in Fig. 6f. A good connection without loss of resistance was observed, even during stretching as shown in Figure S11.

We have designed and fabricated three types of stretchable textile including a rotated woven textile, a knitted textile and a cylindrical textile with a simple encapsulation process with dipping and coating method using silicone rubber. These textiles exhibit excellent stretching performance, stable conductivity under deformation and simple electrical connection to devices or modules by soldering. These stretchable textiles can be used in any application that requires a stretchable interconnection due to exposure to an external deformation environment. These textiles could also be applied in commercial fields with further development since they can be manufactured at low cost using continuous manufacturing machines.

## Conclusion

In this study, various stretchable textiles were introduced and fabricated, including a rotated woven textile, a knitted textile and a cylindrical textile, with a simple encapsulation process using silicone rubber. Cu wires were used for interconnection between electronic devices or modules. The rotated woven textile became stretchable by rotation of the textile at 45° to create a rhombic space between the fabric intersections. This fabric exhibited 67% strain and stable conductivity. The knitted textile also exhibited stretchability, but this was limited by the height of the fabric loop and manufacturing disadvantages. These two types of stretchable textile can be applied to integration of devices within a substrate. A cylindrical textile was also introduced and fabricated using a braiding technique incorporating a fiber rotation mechanism to create a rhombic area between intersections for high stretchability and strength. This textile exhibited a strain of more than 700% and highly stable conductivity under repeated stretching deformation. The textile was used as an electrical interconnection between solar cells and an LED without loss of resistance, and maintained its performance even during stretching. It is expected that this highly stretchable textile can be applied to devices and modules as an interconnection and can be scaled up in both length and area for commercial use.

## Experimental details

### Fabrication stretchable textile interconnection

A commercial copper (Cu) mesh (120Mesh, 0.131 mesh size, Hyunjinmeshfilter) with the smallest available mesh size was used. The weaving process was conducted using a Jacquard machine (Daesung High Tech) and Cu wires (100 μm) were used for both the warp and weft to fabricate a highly stretchable woven textile. The knitted textile was a commercially-available Cu knitted mesh (single wire structure, CKM-01, Boegger). The cylindrical textile was fabricated using a braiding machine (Daesung High Tech) with 12 rotating Cu wires (100 μm). A range of cylindrical textiles with different core diameters was fabricated, and plastic tubing was used for the core.

### Interconnection and integration devices

The fabricated stretchable textiles were encapsulated in silicone rubber (ecoflex-0020 A/B, Smooth-On). Silicone rubber A and B were mixed in a 1:1 (v:v) ratio and the embedded textiles were cured in an oven at 70 °C for 30 min. The edges of the stretchable textile interconnections were soldered using Pb-free wire (HSE-02-SR34, Heesung Material LTD.) and a soldering iron (FX-951, Hakko). Passivated emitter and rear cells (PERC, LWM5BB, Lightway) and light-emitting-diode (LED) chips were connected to the stretchable textile interconnection by soldering.

### Characterization

The tensile properties of the stretchable textile interconnection were measured using a universal mechanical testing machine (UTM, AGS-X, Shimadzu) at a crosshead speed of 100 mm/min. We conducted tensile test with 6 samples that fabricated with same processes. Field-emission scanning electron microscopy (FE-SEM; Hitachi S4800) was performed to observe the woven textile structure.

The electrical conductivity and resistance change of the stretchable textile interconnections were measured using a Keithley 2636B Sourcemeter connected to a custom-built automatic stretching tester (SnM). The photovoltaic performance of the solar cells and arrays was measured by first calibrating a solar simulator (Sun 2000, 1000 W Xenon source; Abet Technologies; 2400 Keithley source meter) with a KG-3 filter and an NREL-certified reference cell, and then setting the simulator to 1 sun, 1.5 AM conditions.

## Supplementary Information

Supplementary Information.
