# Supplementary material for: Highly stretchable large area woven, knitted and robust braided textile based interconnection for stretchable electronics
Source: Sci Rep. 2021 Feb 17;11:4038. doi: 10.1038/s41598-021-83480-x (PMC7890051; doi:10.1038/s41598-021-83480-x)
Supplement: Supplementary file 1 — Supplementary Information. [file 41598_2021_83480_MOESM1_ESM.docx]

**Highly Stretchable Large Area Woven, Knitted and Robust Braided Textile Based Interconnection for Stretchable Electronics**

**Min Ju Yun^1^, Yeon Hyang Sim^1,2^, Dong Y. Lee^1^ and** **Seung I. Cha*^1,2^**

**1. Energy Conversion Research Center, Electrical Materials Research Division, Korea Electrotechnology Research Institute**

**2. Department of Electro-functionality Materials Engineering, University of Science and Technology**

**Supporting Information**


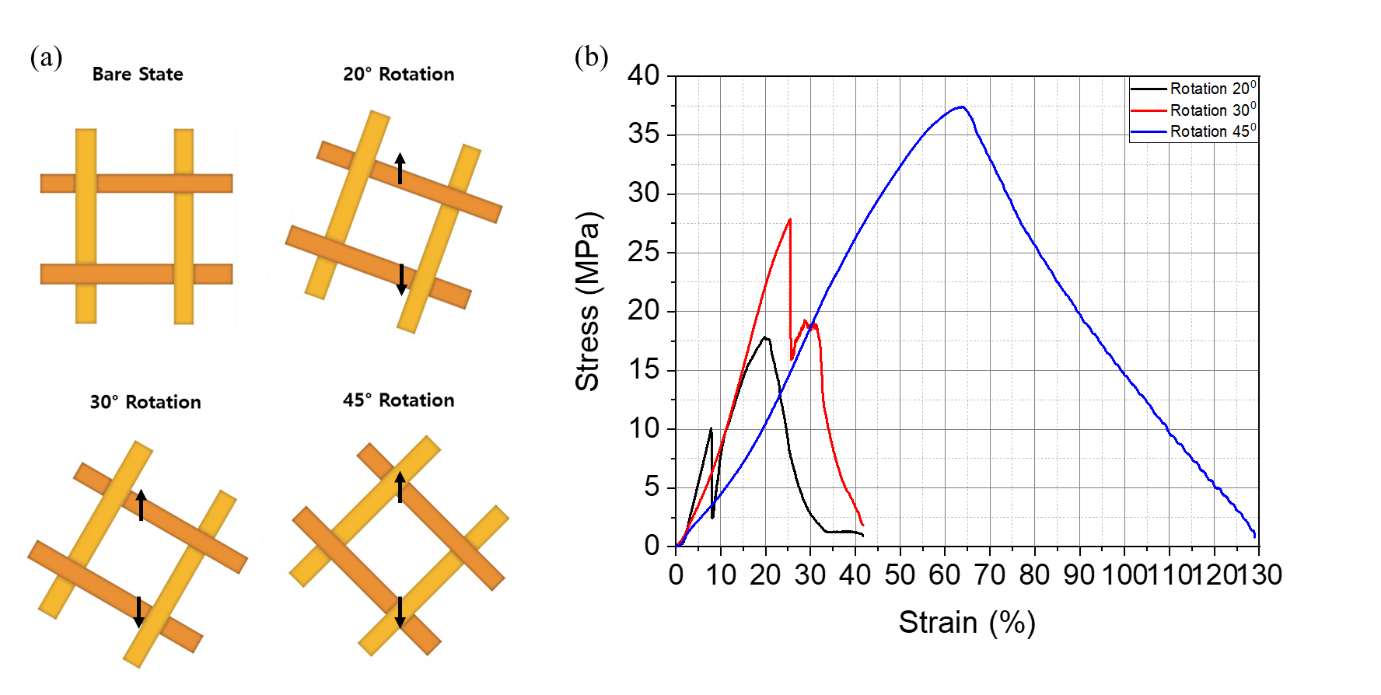


**Figure S1.** (a) Schematic illustration and (b) relationship between strain and stress of rotated woven textile to 20, 30 and 45 degrees.

**
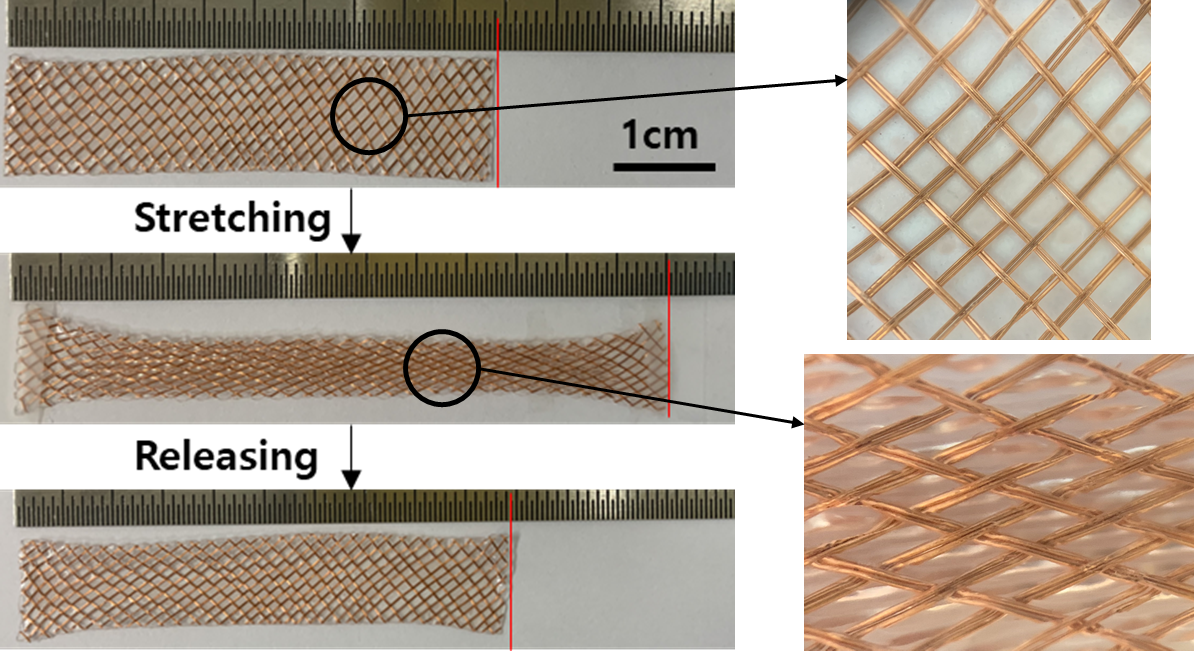
**

**Figure S2.** Photographs of rotated widely woven textile’s stretching and recovering state and enlarge structure images of before stretching and during stretching.

**
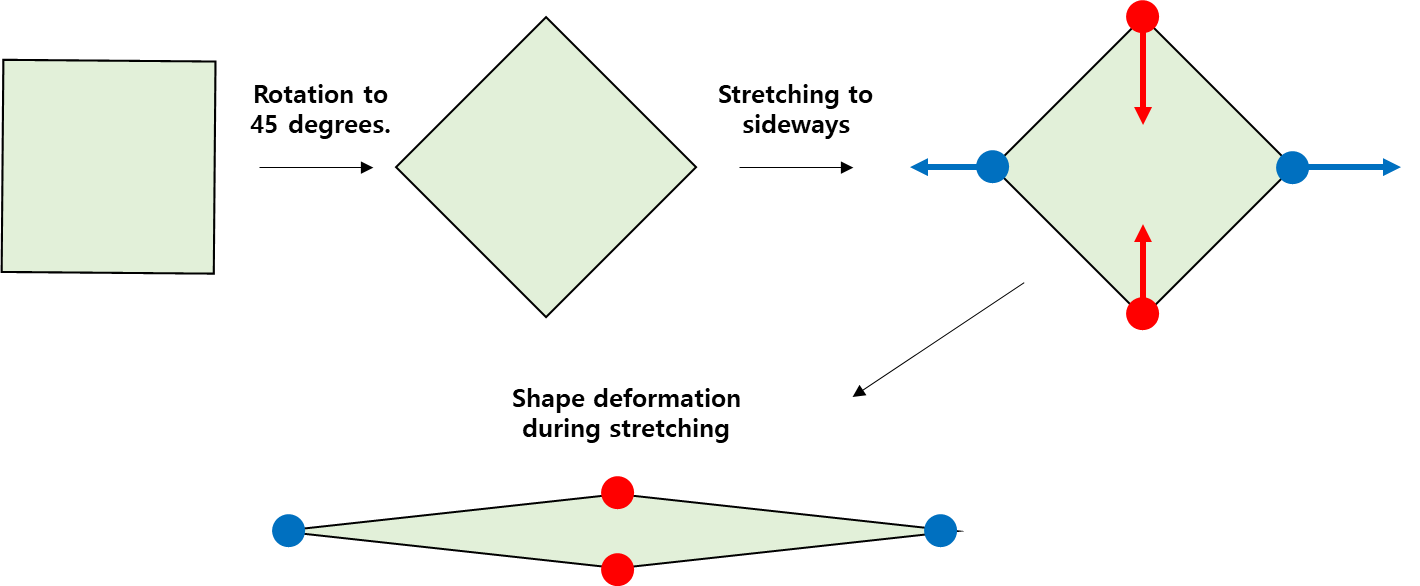
**

**Figure S3.** Schematic illustration intersection space shape changing square to rhombic shape and shape deformation during stretching of woven textile.


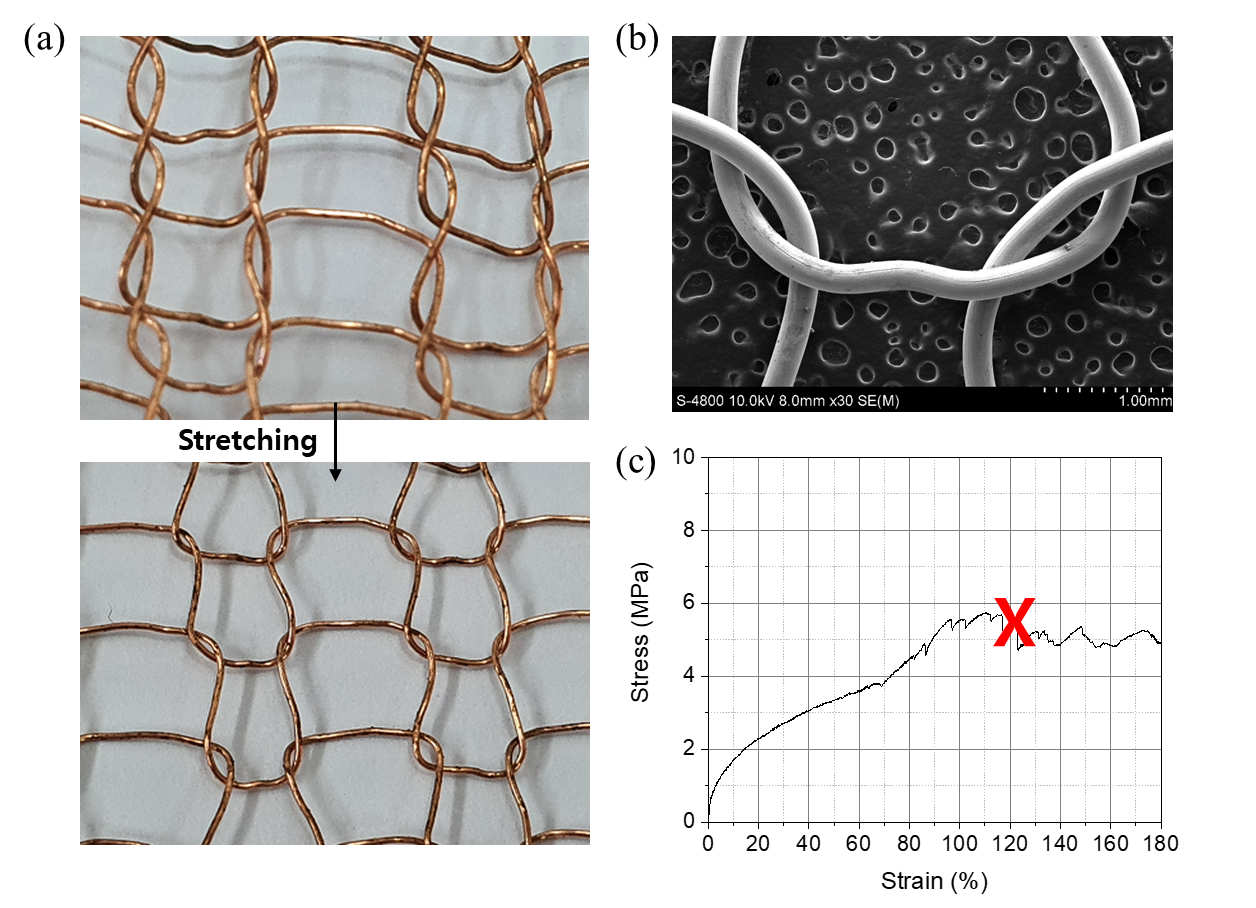


**Figure S4.** (a) Photographs of knitted textile’s stretching and recovering state. (b) SEM image of intersection of loops in knitted textile. (c) Relationship between strain and stress of embedded stretchable knitted textile interconnection.

**Figure S5.** Temperature and repetitive strain times relationship of wide woven textile during 500 cycles of repetitive stretching to 60% strain.


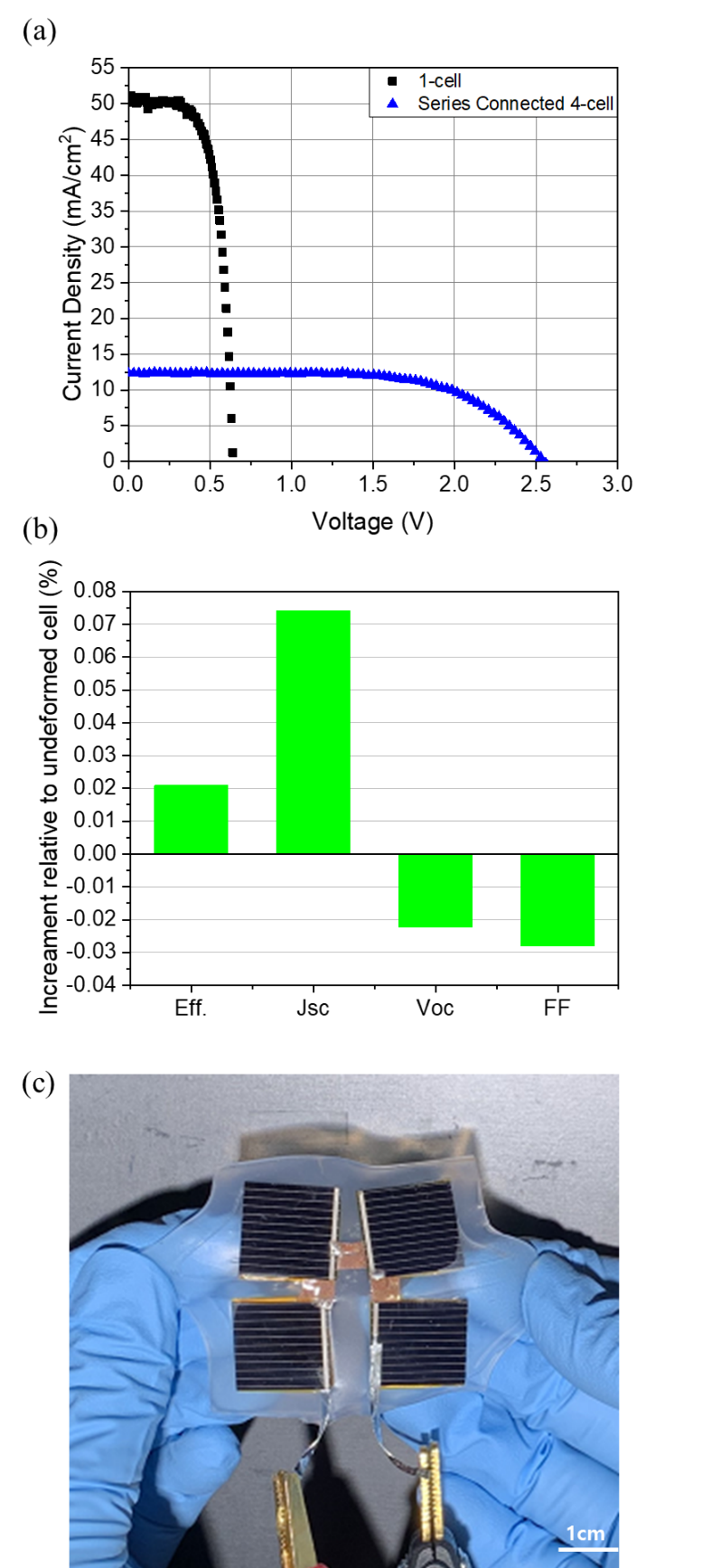


**Figure S6.** (a) Photovoltaic performance of 1-cell and 4-cell series connected module using stretchable textile interconnection and (b) comparing performance before and after stretching. (c) Photograph of measurement solar cell performance under 1 Sun illumination condition.

**
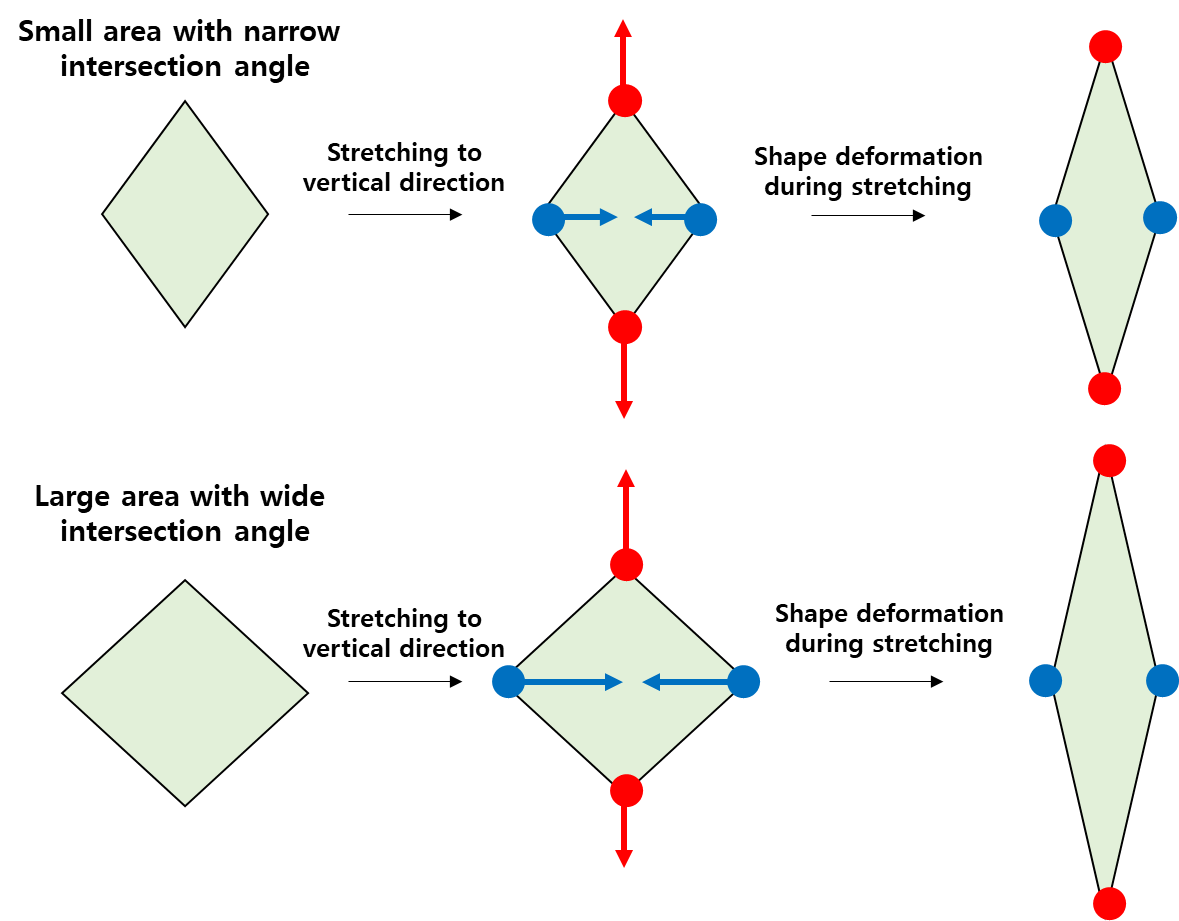
**

**Figure S7.** Schematic illustration shape deformation during stretching of cylindrical textile depends on intersection angle.

**
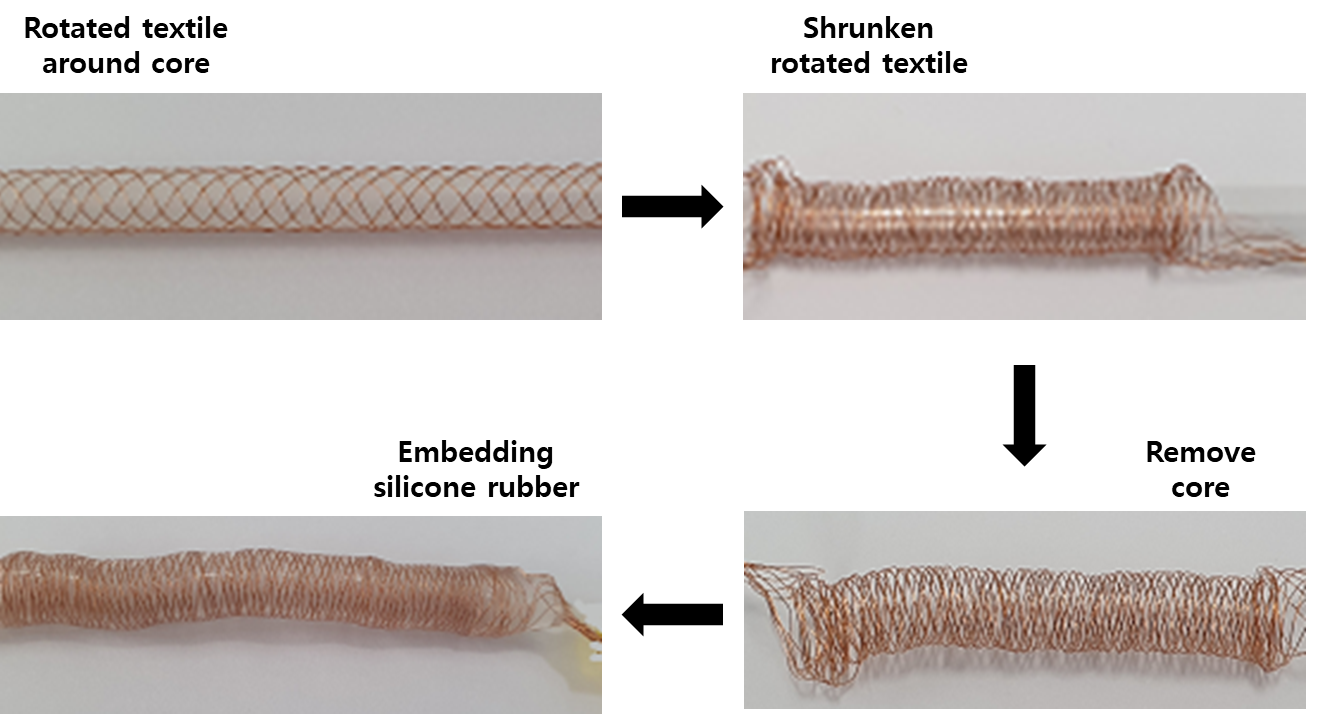
**

**Figure S8.** Photographs of fabrication process of stretchable cylindrical textile interconnection from fibers rotation around core to embedding in silicone rubber.


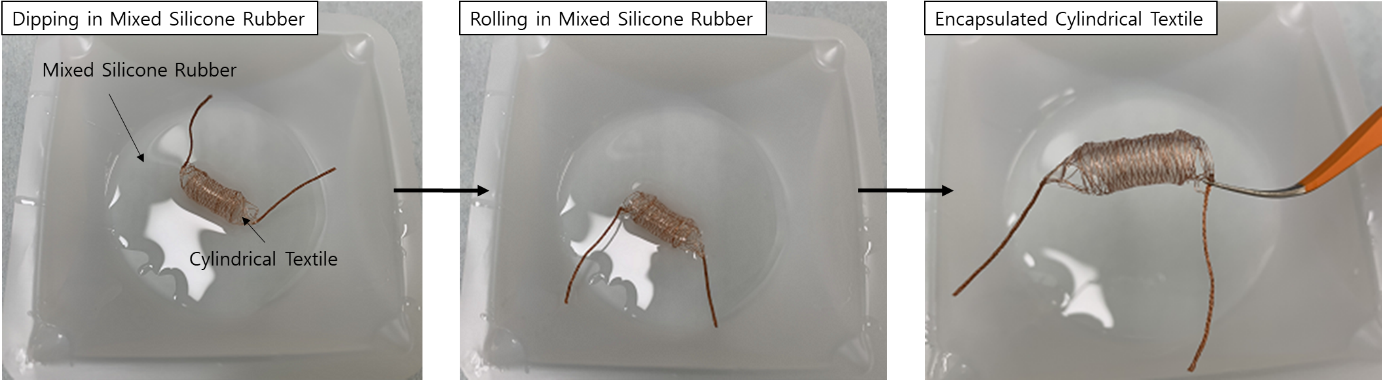


**Figure S9.** Photographs of encapsulation process of stretchable cylindrical textile interconnection by dipping and rolling in mixed silicone rubber and forming encapsulation layer before curing silicone rubber.


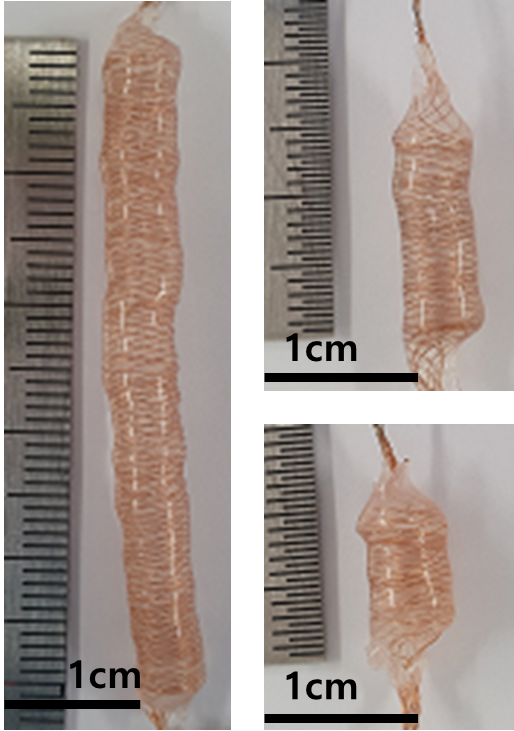


**Figure S10.** Photographs of various length of stretchable cylindrical textile interconnection.


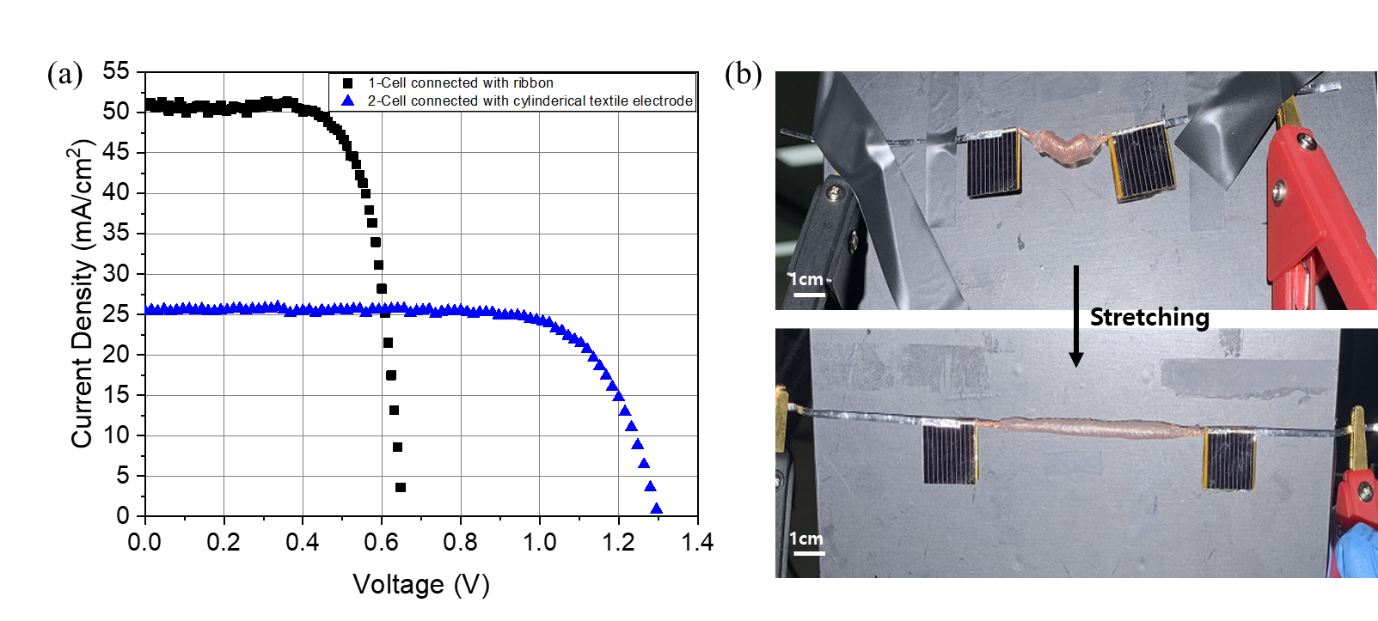


**Figure S11.** (a) Photovoltaic performance of 2- solar cells series connected using stretchable cylindrical textile interconnection. (b) Photograph of 2-solar cells connected and stretched with cylindrical textile interconnection.
